# Kidney dynamic SPECT acquisition on a CZT swiveling-detector ring camera: an in vivo pilot study

**DOI:** 10.1186/s12880-024-01271-y

**Published:** 2024-04-22

**Authors:** Michel Hesse, Florian Dupont, Nizar Mourad, Pavel Babczenko, Gwen Beaurin, Daela Xhema, Eliano Bonaccorsi-Riani, François Jamar, Renaud Lhommel

**Affiliations:** 1 Nuclear Medicine Department, Cliniques Universitaires Saint-Luc, 10 Avenue Hippocrate, 1200 Brussels, Belgium; 2 Pôle de Chirurgie Expérimentale et Transplantation– CHEX, Cliniques Universitaires Saint-Luc, Brussels, Belgium

**Keywords:** Dynamic, SPECT, CZT, Ring camera, MAG3, Kidney

## Abstract

**Background:**

Large field of view CZT SPECT cameras with a ring geometry are available for some years now. Thanks to their good sensitivity and high temporal resolution, general dynamic SPECT imaging may be performed more easily, without resorting to dedicated systems. To evaluate the dynamic SPECT imaging by such cameras, we have performed an in vivo pilot study to analyze the kidney function of a pig and compare the results to standard dynamic planar imaging by a conventional gamma camera.

**Methods:**

A 7-week-old (12 kg) female Landrace pig was injected with [^99m^Tc]Tc-MAG3 and a 30 min dynamic SPECT acquisition of the kidneys was performed on a CZT ring camera. A fast SPECT/CT was acquired with the same camera immediately after the dynamic SPECT, without moving the pig, and used for attenuation correction and drawing regions of interest. The next day the same pig underwent a dynamic planar imaging of the kidneys by a conventional 2-head gamma camera. The dynamic SPECT acquisition was reconstructed using a MLEM algorithm with up to 20 iterations, with and without attenuation correction. Time-activity curves of the total counts of each kidney were extracted from 2D and 3D dynamic images. An adapted 2-compartment model was derived to fit the data points and extract physiological parameters. Comparison of these parameters was performed between the different reconstructions and acquisitions.

**Results:**

Time-activity curves were nicely fitted with the 2-compartment model taking into account the anesthesia and bladder filling. Kidney physiological parameters were found in agreement with literature values. Good agreement of these parameters was obtained for the right kidney between dynamic SPECT and planar imaging. Regional analysis of the kidneys can be performed in the case of the dynamic SPECT imaging and provided good agreement with the whole kidney results.

**Conclusions:**

Dynamic SPECT imaging is feasible with CZT swiveling-detector ring cameras and provides results in agreement with dynamic planar imaging by conventional gamma cameras. Regional analysis of organs uptake and clearance becomes possible. Further studies are required regarding the optimization of acquisition and reconstruction parameters to improve image quality and enable absolute quantification.

**Supplementary Information:**

The online version contains supplementary material available at 10.1186/s12880-024-01271-y.

## Background

In nuclear medicine, metabolic processes are best studied using dynamic imaging. While single photon emission computed tomography (SPECT) imaging enhances the diagnostic quality compared to 2D imaging, performing dynamic SPECT has been hindered by the design of conventional gamma cameras [1]. Indeed, SPECT imaging requires to rotate the gamma camera detectors around the patient to get sufficient data to reconstruct the 3D activity distribution of the radiopharmaceutical injected to the patient. The rotation time thus limits the temporal resolution achievable in dynamic SPECT.

Fast rotating systems may complete tomographic acquisitions in 10s or less but suffers from a low sensitivity, somewhat compensated by using more detector heads. To palliate inconsistencies between the angular SPECT data when camera rotation is slower than studied processes, techniques to evaluate kinetic model parameters directly from projection data have been developed [1]. Other approaches introduce temporal or spatial constraints in the SPECT reconstruction of the dynamic data to improve the images with “slow”-rotation dynamic SPECT [2, 3].

On the other hand, dedicated systems have been developed to get rid of the detector rotations by placing multiple detectors around the patient and acquiring all tomographic views simultaneously. For cost reasons, most of these systems had a small field of view (FOV) and were limited to small organs such as the brain or the heart [4–6].

Since more than a decade now, Cadmium-Zinc-Telluride (CZT)-based cameras are available. Thanks to their compactness, these digital detectors can be tilled up in a camera to surround (part of) the patient. Initially, such CZT systems were dedicated to cardiac studies [5, 6] but more recently large FOV SPECT cameras with a ring geometry have become available [7, 8]. Thanks to the detector ring geometry, lower temporal resolutions (down to 1s) become possible. Data are collected from all around the patient simultaneously by 12 column detectors and the temporal sampling is only limited by the time needed by all detectors to scan the patient area with their swiveling motion.

The present study aims at analyzing the dynamic SPECT acquisition mode of such a CZT swiveling-detector ring camera. We have performed an in vivo pilot study of the kidney function of a pig with such a camera and compared the results to those obtained with standard planar dynamic imaging of the same animal performed on a conventional 2-head gamma camera.

## Materials and methods

### Animal

A 7-week-old (12 kg) female Belgian Landrace pig was used for the experiment. The study was authorized by the institutional ethics committee related to experimentation on animals (ref. 2022/UCL/MD/052 approved on the 4th of April 2023).

Anesthesia was performed just before the start of image acquisition and was induced via intramuscular injection of 6 mg/kg Tiletamine 50 mg/ml and Zolazepam 50 mg/ml (Virbac, Leuven, Belgium) and 2 mg/kg Xylazine 2% (Bayer, Mechelen, Belgium) and maintained during the realization of the experiment by two intravenous injections of Zolazepam through a 18G intravenous catheter inserted in an ear vein just after anesthesia induction.

### Acquisitions

The dynamic SPECT was performed on a General Electric Starguide SPECT/CT (GE Healthcare, Haifa, Israel). This camera benefits from 7.3 mm-thick CZT crystals equipped with Tungsten parallel-hole collimators. Four square collimator holes (1.03 × 1.03mm^2^) are associated to each CZT pixel (2.46 × 2.46mm^2^). The pig was installed in supine position on the camera table so that its kidneys were in the camera FOV. The 30 min dynamic acquisition was started a few seconds before the injection of 77.7MBq of [^99m^Tc]Tc-MAG3 through a catheter inserted into the pig’s ear. The delay between the acquisition’s start and the injection time was set to allow the system to position the detectors close to the animal after the start command initiation. The detector swivel motion was set to scan the pig area in 5s. Raw data were saved into a list mode file. As the current software version of the Starguide camera does not allow to perform a CT scan in the same workflow than the dynamic SPECT, we have performed a static SPECT/CT immediately after the dynamic SPECT acquisition in order to get a CT for attenuation correction.

The next day, another study of the kidneys of the same pig was performed as a planar dynamic acquisition on a Philips Brightview 2-head gamma camera (Philips, Milpitas, CA). This camera includes a 0.95 cm-thick NaI crystal associated with a LEHR parallel-hole collimator. The energy window was set as 140.51 keV ± 10%. The 30 min dynamic planar acquisition was recorded, in a 64 × 64 pixels matrix, as 60 frames of 5s followed by 150 frames of 10s. The temporal sampling was therefore similar to the one of the SPECT acquisition. The acquisition was started while injecting 77.7MBq of [^99m^Tc]Tc-MAG3.

For both acquisitions, the [^99m^Tc]Tc-MAG3 solution had a volume of about 2 ml and was injected as a bolus, followed by a flush of saline solution to rinse the tubing.

The pig was sedated before each acquisition and the sedation was controlled and adjusted during the acquisition to reduce any animal motion.

### SPECT reconstructions

The list mode file of the dynamic SPECT acquisition was sorted to generate a set of projections for each of the 12 column detectors, by grouping the continuous swivel angles in 2 degrees bins, i.e., the factory sampling for routine static SPECT acquisitions. The resulting numbers of projections vary from detector to detector according to the swivel range needed to cover the pig area from each detector position. They were equal to 55, 48, 36, 50, 68, 74, 71, 62, 32, 57, 55 and 58 for the 12 detectors in clock order, respectively. Native pixel size was used for the projections, i.e., 2.46 × 2.46 mm in a 16 × 112 matrix, corresponding to the 7 CZT modules (4 × 4cm^2^) of each column detector. The energy window was set as 140.51 keV ± 10%. To evaluate the temporal sampling, the 30 min acquisition was split up as 360 frames of 5s (360 × 5s), 180 frames of 10s (180 × 10s), 120 frames of 15s (120 × 15s), 60 frames of 30s (60 × 30s) and 30 frames of 60s (30 × 60s). Each time frame was then reconstructed using a Maximum Likelihood Expectation Maximization (MLEM) algorithm. To analyze the convergence of the reconstruction algorithm, up to 20 iterations were carried out on the 60 × 30s reframing, which is less impacted by Poisson noise in the projections. Reconstructions were performed with and without CT-based attenuation correction to produce computed tomography attenuation corrected (CTAC) and non-attenuation corrected (NAC) images, respectively. The registration between the CT and the dynamic SPECT resulted from the table positions saved in the Digital Imaging and COmmunications in Medicine (DICOM) header of both acquisitions, as the pig was not moved between the scans.

### Data analysis

Regions of interest (ROI) were manually drawn around both kidneys and the background on the planar images. Time-activity curves (TAC) were generated by correcting the total counts of each kidney ROI for background activity.

For the SPECT images, ROIs were manually drawn around the kidneys on the CT image, then transferred to the registered dynamic SPECT images. To compensate for partial volume effects and limited SPECT spatial resolution, the CT ROIs have to be extended on the SPECT image. A comparison of the dynamic curve parameters was performed for extensions of the ROIs from 0 cm up to 2 cm in all directions. Total counts of each kidney ROI in NAC and CTAC images were compiled into corresponding TACs. To illustrate one of the advantages of dynamic SPECT over dynamic planar imaging, i.e., the possibility to perform regional analysis of the organs, the kidney’s ROIs were split in halves along the superior-inferior axis. These kidney’s halves were then processed separately to generate TACs to be compared to the full kidneys TACs.

### Compartmental model

To analyze the time-activity curves, we use an adapted 2-compartment kinetic model. In the 2-compartment model, the activity concentration in the kidney ${C}_{K}\left(t\right)$ can be expressed as a bi-exponential function

1 $$ {C}_{K}\left(t\right)=A\left({e}^{-{k}_{o}t}-{e}^{-{k}_{i}t}\right)$$

with the rate constants $$ {k}_{i}$$ and $$ {k}_{o}$$corresponding to the transfers from the blood to the kidney and from the kidney to the bladder, respectively.

Because the pig did not empty its bladder neither before nor during the scans, and as no catheter was introduced into the bladder to force its emptying, we cannot expect a constant rate of elimination of the MAG3 from the kidneys [9, 10]. The sedative injected to the animal may also impact the constant rate $$ {k}_{o}$$ [11]. To take these effects into account in the model, we introduce a time dependent rate $$ {K}_{o}\left(t\right)$$, and Eq. (1) becomes

2 $$ {C}_{K}\left(t\right)=A\left({e}^{-{\int }_{0}^{t}{K}_{o}\left({t}^{{\prime }}\right)d{t}^{{\prime }}}-{e}^{-{k}_{i}t}\right)$$

As the ${K}_{o}\left(t\right)$ function is unknown and includes many animal- and scan-dependent parameters, we make the assumption that it can be approximated by an exponential function as

3 $$ {K}_{o}\left(t\right)={k}_{o}{e}^{-\beta t}$$

where $$ {k}_{o}$$ and β are parameters. Expression (3) has the expected boundary behavior: at t = 0, we find the initial constant rate factor $$ {k}_{o}$$when the bladder is empty, and at larger times, $$ {K}_{o}\left(t\right)$$ tends to zero as expected when the bladder is full. Equation (3) includes the normal case of no bladder blocking when β is equal to zero.

Combining Eqs. (2) and (3), we get the adapted 2-compartment model for the kidney function

4 $$ {C}_{K}\left(t\right)=A\left({e}^{-\frac{{k}_{o}}{\beta }\left(1-{e}^{-\beta t}\right)}-{e}^{-{k}_{i}t}\right)$$

where the parameters $$ A$$, $$ {k}_{o}$$, β and $$ {k}_{i}$$ have to be fitted to the measured time-activity curves.

## Results

**Fig. 1:**
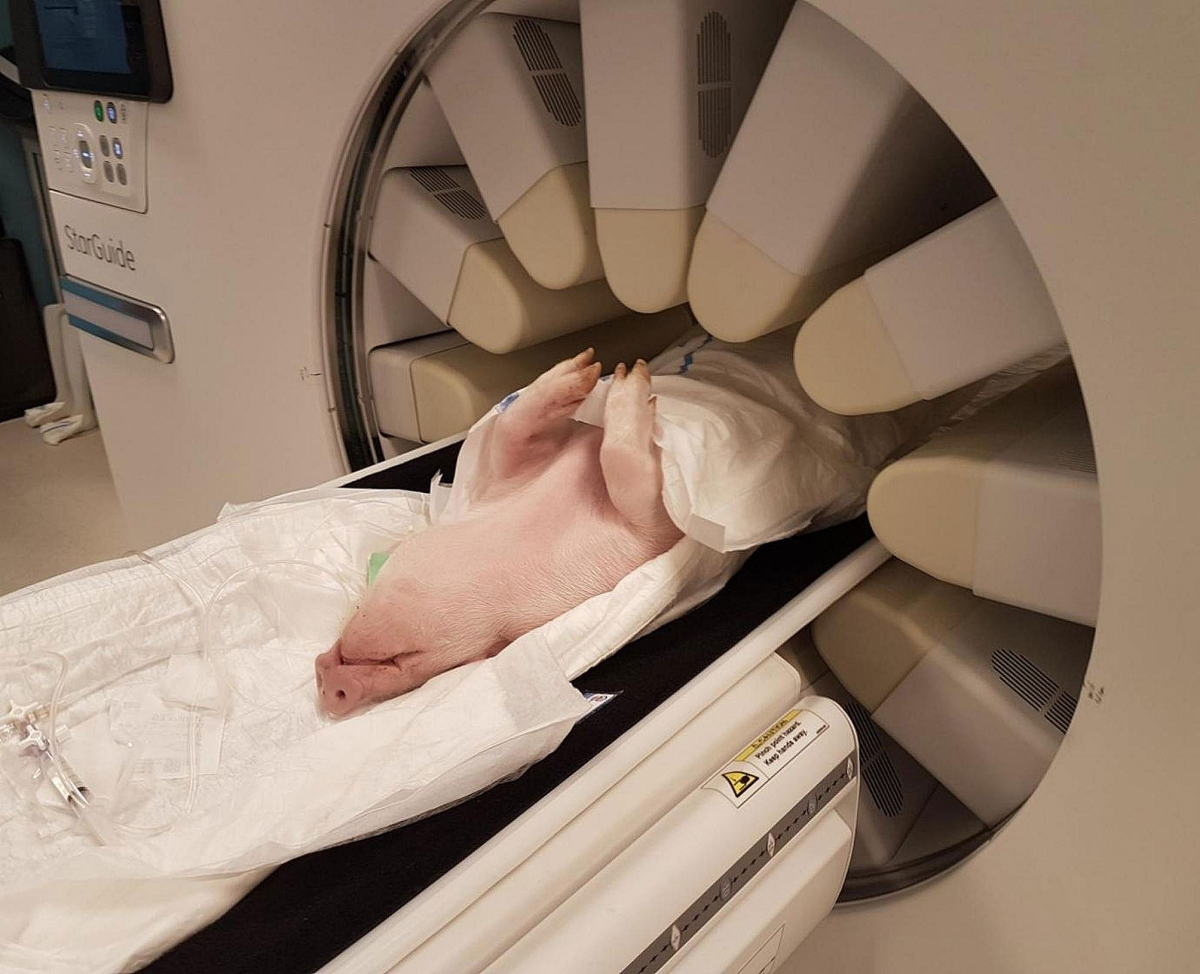
Picture of the pig during the dynamic SPECT acquisition with the Starguide camera, illustrating the static radial and angular positions of the 12 detectors during the scan

The Fig. 1 illustrates the positioning of the pig on the Starguide system during the dynamic SPECT acquisition. The sedated animal was lying on the camera bed in supine position with its kidneys in the camera FOV. The 12 column detectors came as close as possible to the animal according to an optical contour scan performed at initial positioning step. Minimal radial positions of the lateral and posterior detectors were constrained by the camera bed while the pig’s posterior legs restricted anterior detectors radii. The radial and angular positions of the 12 detectors are fixed during the dynamic scan: the only motion performed is the swivel of each column detector behind its cover.

**Fig. 2:**
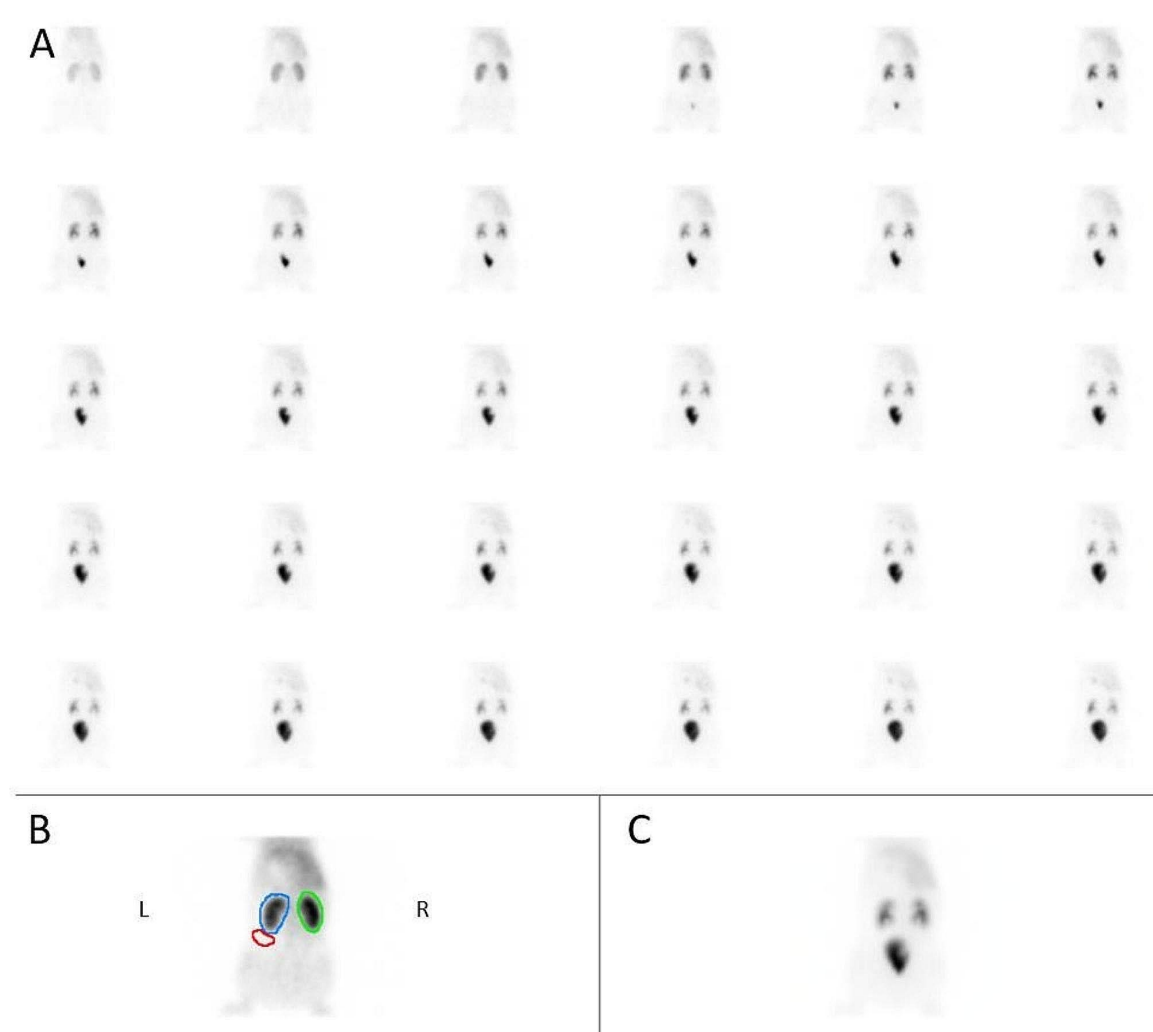
Dynamic planar images of the pig by the conventional gamma camera. **A** The 30 min dynamic acquisition was reframed as 30 images of 60s. **B** Summed image of the first 3 min of the dynamic planar acquisition, illustrating the uptake phase, together with left (blue) and right (green) kidneys and background (red) ROIs. **C** Summed image of the full acquisition, illustrating the pelvic phase and the bladder filling

Resulting images from the dynamic planar acquisition are displayed in Fig. 2. For display clarity, the planar acquisition was reframed as 30 images of 60s that are all shown in Fig. 2A. The large FOV of the Philips Brightview gamma camera (54 cm) enables the acquisition of the liver, kidneys and bladder in one image. Reframing the dynamic acquisition allows to highlight the uptake and pelvic phases as illustrated in Fig. 2B with the sum of the acquisition’s first 3 min, and in Fig. 2C with the sum of the full acquisition, respectively. The image in Fig. 2B was used to manually draw the kidneys and background ROIs to generate the time-activity curves.

**Fig. 3:**
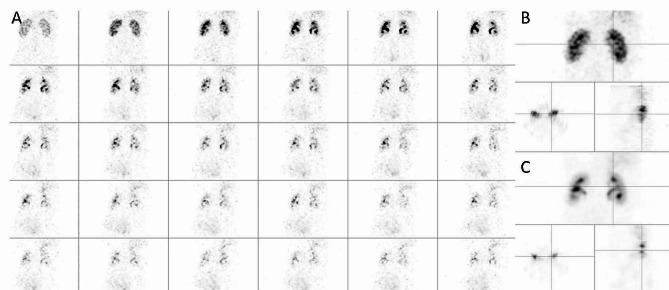
Dynamic NAC SPECT image of the pig reconstructed from the Starguide acquisition. **A** Same coronal slice of all 60s frames of the 30 min dynamic acquisition. **B, C** Coronal, axial and sagittal views of the SPECT images reconstructed from the acquisition’s first 3 min and all 30 min, respectively

The reconstructed NAC images from the dynamic SPECT acquisition are illustrated in Fig. 3 and correspond to 20 MLEM iterations. To ease the comparison with the dynamic planar images from Fig. 2, a similar layout was used. Figure 3A presents the same coronal slice from all NAC SPECT images corresponding to the 30 frames of 60s that span the dynamic SPECT acquisition. Of course, each view of Fig. 3A is associated to a 3D image as illustrated by Fig. 3B and C, that present the coronal, axial and sagittal views of the NAC SPECT images of the first 3 min and the full 30 min acquisition, respectively. Note that, as the Starguide axial FOV is only about 28 cm, it is more difficult to get the liver, the kidneys and the bladder in the same image.

**Fig. 4:**
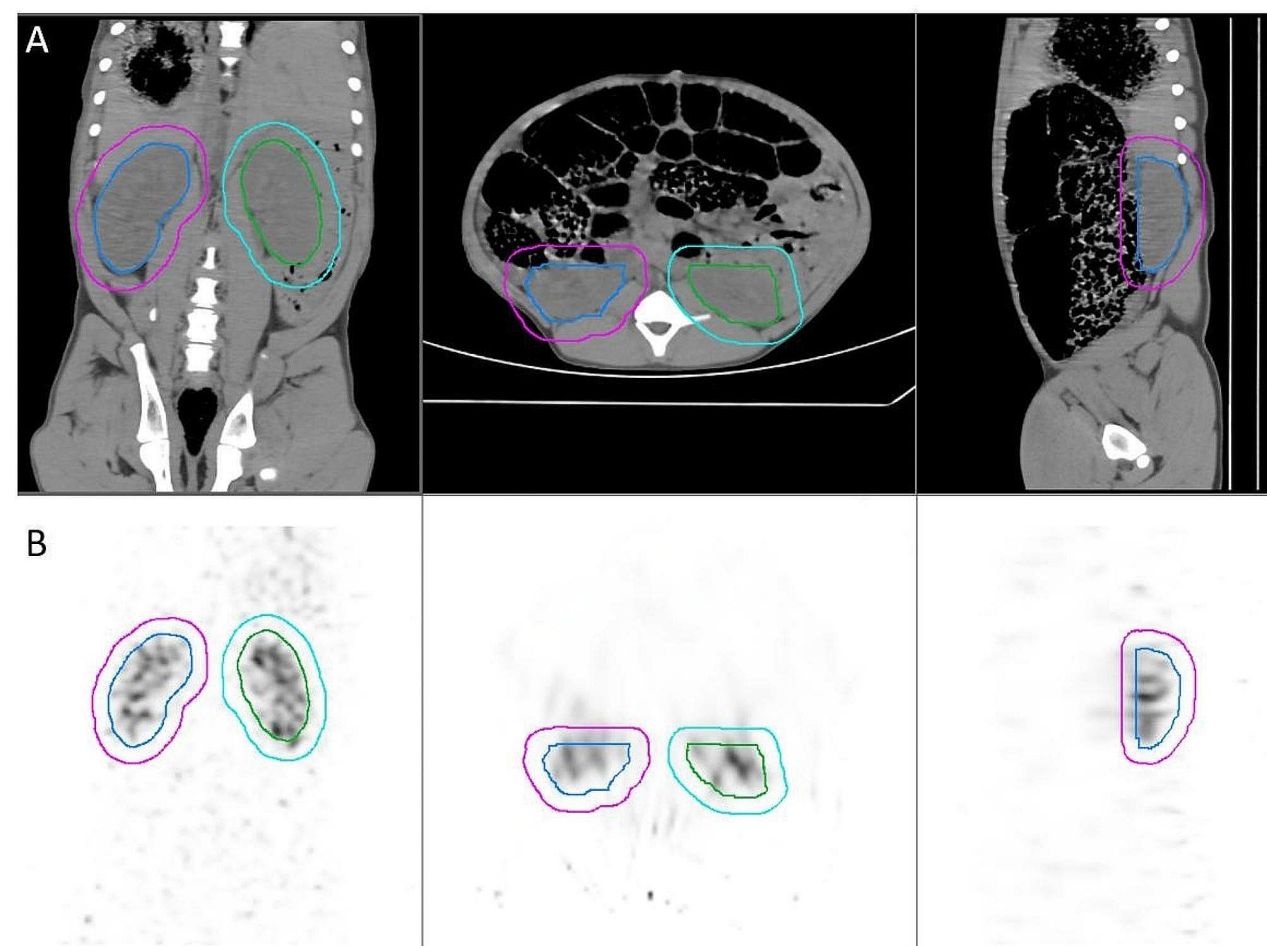
A CT image of the pig by the Starguide. ROIs were manually drawn around the left (blue) and right (green) kidneys. Final SPECT ROIs were obtained by extending the CT ROIs, as illustrated by the left (purple) and right (cyan) kidney’s ROIs extended by 1 cm in all directions. **B** NAC SPECT image corresponding to the fourth 30s frame reconstructed with 20 MLEM iterations, with kidney’s ROIs

Figure 4 shows the kidney’s ROIs used to generate TACs for the dynamic SPECT acquisition. Initial and 1 cm-extended ROIs are displayed on the CT image in Fig. 4A. These ROIs are transferred to the dynamic SPECT image, naturally registered to the CT, as illustrated in Fig. 4B on the fourth 30s SPECT frame. When looking at dynamic curves’ global parameters, like the clearance halftime or the time-to-peak, we observed only small variations of less than 5% for ROIs with an extension between 0.3 and 1.7 cm in all directions. The time-to-peak is the time from the bolus injection to the TAC maximum, and the clearance halftime is the time for the kidney total counts to be divided by two after reaching its maximum value. In the following, we will use the 1 cm extension of the CT kidney ROIs to derive TACs.

**Fig. 5:**
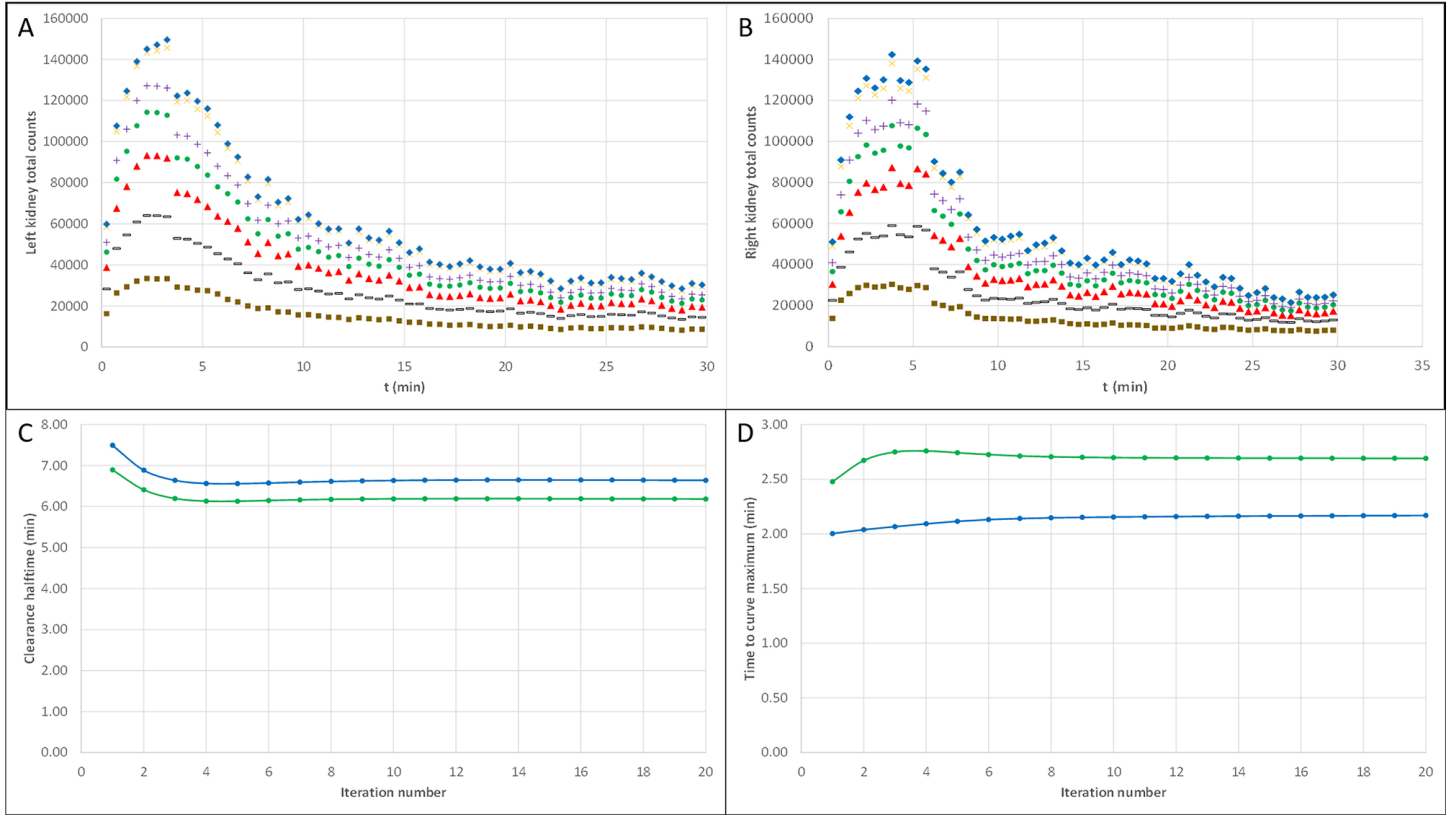
Convergence of the MLEM reconstruction of the 60 × 30s projections with respect to the iteration number. **A** Dynamic curves of the left kidney total counts for 1 (brown squares), 2 (black dotted lines), 3 (red triangles), 4 (green bullets), 5 (purple plus), 10 (orange crosses) and 20 (blue diamonds) iterations. **B** Same as A for the right kidney total counts. **C** Clearance halftime evaluated from the dynamic curves for the left (blue line) and right (green line) kidneys as a function of the iteration number. **D** Time-to-peak of the dynamic curves for the left (blue line) and right (green line) kidneys as a function of the iteration number

The convergence of the MLEM reconstruction is illustrated, with the 60 × 30s reframing and CT ROIs extended by 1 cm, in Fig. 5. Figure 5A and B show that kidneys total counts converge with the number of iterations. MLEM reconstructions using 20 iterations provide ROIs total counts with an error of less than 0.1% for all image frames. Reaching such an accuracy on physiological parameters requires less iterations. The time-to-peak and the clearance halftime only require 7 iterations to get an error of about 1% (see Fig. 5C and D). In the following, all presented results for the 3D dynamic acquisition will correspond to 20 MLEM iterations.

**Fig. 6:**
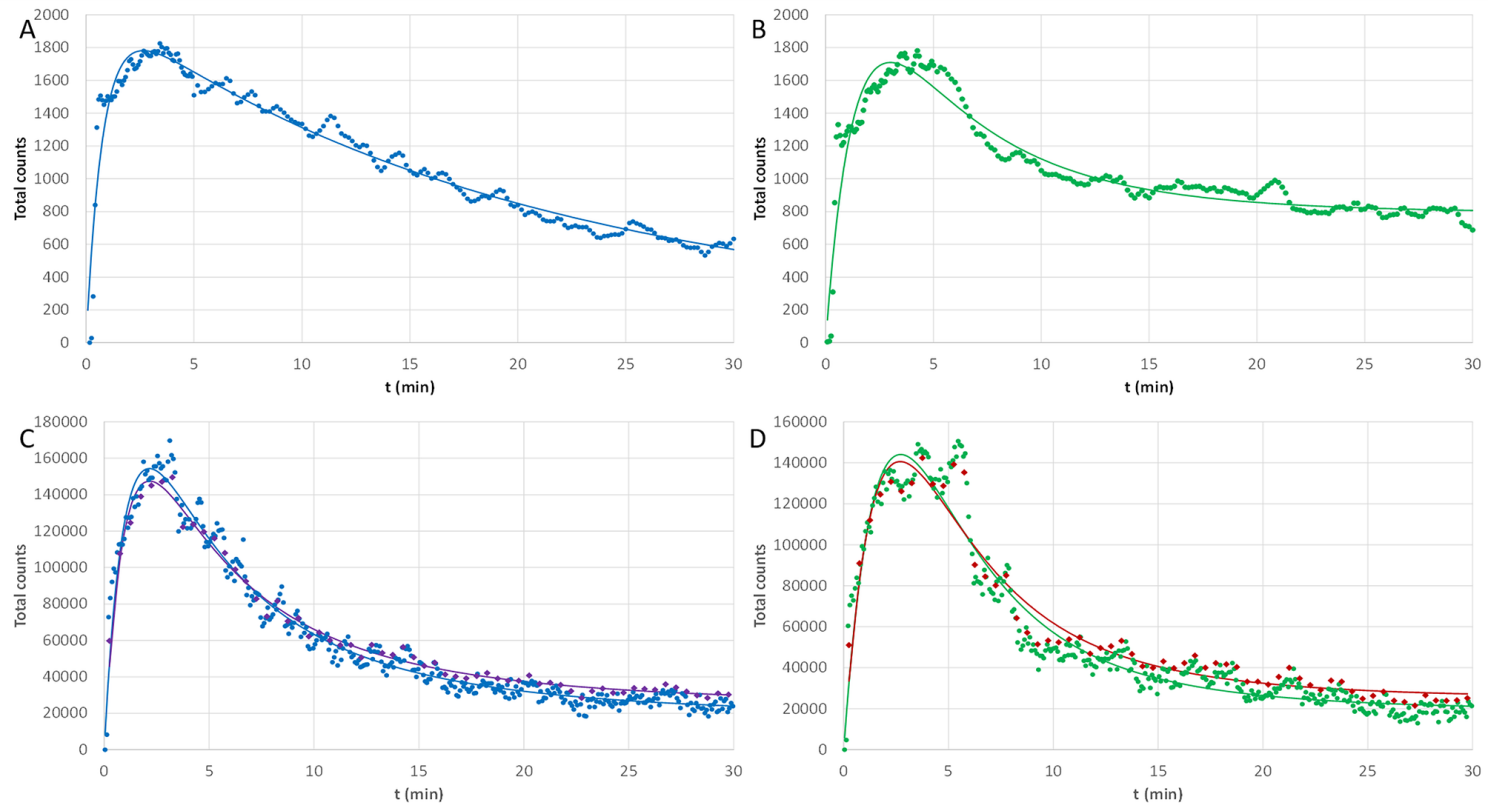
TACs for the different dynamic acquisitions and reconstructions. **A, B** Total counts from the planar acquisition for the left and right kidneys, respectively. **C** TACs for the left kidney from the NAC images for the 360 × 5s (blue bullets) and the 60 × 30s (purple diamonds) reframings of the dynamic SPECT acquisition, respectively. **D** TACs for the right kidney from the NAC images for the 360 × 5s (green bullets) and the 60 × 30s (red diamonds) reframings of the dynamic SPECT acquisition, respectively. Full lines correspond to the theoretical fits of the data points

The TACs of the left and right kidneys from 2D and 3D dynamic images are plotted in Fig. 6. Figure 6A and B correspond to the left and right kidneys from the dynamic planar acquisition, respectively. Two sets of curves, generated from the dynamic SPECT acquisition using the kidneys ROIs on the NAC image for the 360 × 5s and the 60 × 30s reframings, are displayed in Fig. 6C and D for the left and right kidneys, respectively. In all cases, the measured data points were fit using expression (4), with the best fitting parameters for each case given in Table 1.

**Table 1:** Parameters of the TACs fits in Fig. 6

Left kidney	2D dynamic	3D NAC 360 × 5s	3D NAC 60 × 30s
A	2104	307,920	280,149
k_o_	0.049	0.234	0.222
k_i_	1.293	0.950	0.978
β	0.008	0.085	0.094
**Right kidney**	**2D dynamic**	**3D NAC 360 × 5s**	**3D NAC 60 × 30s**
A	4004	1,070,355	758,808
k_o_	0.248	0.516	0.449
k_i_	0.697	0.649	0.644
β	0.154	0.129	0.132

Parameters of the TACs fits according to expression (4) for both kidneys and corresponding to the planar acquisition and to the 360 × 5s and 60 × 30s reframings of the dynamic SPECT acquisition

From the theoretical curves fitting the measured TACs in Fig. 6, we can extract some meaningful values for the kidney function analysis such as the time-to-peak and the clearance halftime. These kidney parameters are given in Table 2 for the TACs of the dynamic planar acquisition and all temporal reframings of the dynamic SPECT acquisition.

**Table 2:** Dynamic physiological parameters extracted from the planar and SPECT acquisitions

Left kidney	2D dynamic	3D NAC 360 × 5s	3D NAC 180 × 10s	3D NAC 120 × 15s	3D NAC 60 × 30s	3D NAC 30 × 60s
Time to peak (min)	2.6	2.2	2.1	2.1	2.2	2.2
Clearance halftime (min)	16.2	6.0	6.4	6.5	6.6	6.8
**Right kidney**	**2D dynamic**	**3D NAC 360 × 5s**	**3D NAC 180 × 10s**	**3D NAC 120 × 15s**	**3D NAC 60 × 30s**	**3D NAC 30 × 60s**
Time to peak (min)	3.0	2.7	2.7	2.7	2.7	2.7
Clearance halftime (min)	17.1	5.6	5.9	6.0	6.2	6.2

Time to peak and clearance halftime extracted from the fitted curves of the different TACs corresponding to the NAC images of the 360 × 5s, 180 × 10s, 120 × 15s, 60 × 30s and 30 × 60s reframings of the SPECT acquisition

The TACs corresponding to the CTAC image and to the upper and lower halves of each kidney for the 60 × 30s reframing of the SPECT acquisition are illustrated in supplementary Fig. 1. Associated fit parameters of expression (4) are given in supplementary Table e1, while supplementary Table 2 shows the extracted values for the corresponding time-to-peak and clearance halftimes.

## Discussion

Analysis of dynamic SPECT performed with CZT swivelling-detector ring cameras have already been reported for bone [12] and cardiac studies [13]. As far as we know, the present study is the first to evaluate in vivo the kidney function with such a camera and to compare it to standard dynamic planar imaging of the same subject. The pig positioning in the camera FOV was performed by locating its kidneys positions relatively to the camera bed ruler. Future software release of the GE Starguide should improve dynamic protocols by incorporating an initial CT scan to optimize the positioning. As the axial FOV of the GE Starguide is shorter (about 28 cm) than conventional gamma cameras (more than 40 cm), getting multiple organs, like for example kidneys and bladder, in the same dynamic acquisition becomes more difficult.

A specificity of this camera design is that, once the column detectors radii are set (see Fig. 1), only the swivel motion of each detector is enabled during the acquisition. As this motion is hidden behind the detector covers, the system appears to be static for the patient during the whole acquisition. This may so be less stressful for patients than a fast rotating detector head if the same dynamic SPECT was performed on a conventional gamma camera.

The dynamic SPECT data are stored in a list mode file format, which enables a reframing of the acquisition at selected frame durations, multiple of the swivel temporal sampling. For illustration purpose, similar layouts were used for the dynamic frames in 2D and 3D acquisitions, as shown in Figs. 2 and 3, to evaluate qualitatively the kidney function of the subject. This kind of layout allows the physician to identify the uptake and pelvic phases and see the filling of the bladder. Because dynamic SPECT images contain a lot of slices and frames, it is practically impossible to display them all together in the same layout. Planar images could be simulated by summing the SPECT slices but this would cause a loss of information available in the 3D images. The best layouts, providing all qualitative information in the simplest way, will have to be defined for daily practice of dynamic SPECT.

ROIs can be drawn manually on the SPECT images, like what is usually done for the dynamic planar imaging, but we can also use an associated CT image to get anatomical ROIs. Because of the difference in spatial resolutions of the CT and SPECT images, some margin must be added to cover all kidneys activity on the SPECT images (Fig. 4). Here we have chosen a margin of 1 cm in all directions to avoid any significant activity loss. This roughly corresponds to the camera spatial resolution. We have checked the stability of the extracted dynamic parameters with respect to the ROIs extension: variations of less than 5% were obtained for ROIs extensions between 0.3 and 1.7 cm. Using a too small or no extension produces an underestimation of kidney counts, while a too large extension will add counts from surrounding tissues or organs. The value of the ROIs extension should be evaluated more precisely, for examples with phantoms, as the SPECT resolution is strongly impacted by the angular sampling of the acquisition. Indeed, without the gantry rotation, some angular views of the patient are missing, which has an impact on the quality of the reconstructed image (see artefactual lines visible on the SPECT axial view in Fig. 4) [14].

Comparison of the different TACs illustrated in Fig. 6 provides several interesting results. The TACs extracted from the dynamic SPECT reconstructions have the expected shape for a renogram, with a rapid maximum followed by a slower activity decrease. In the present case, correcting for attenuation has no significant impact on the kidney’s TACs (see supplementary Fig. 1B and 1D). This is not surprising as the pig was young (about 7 weeks old) and weighs only about 12 kg. The impact might be more important in adult patients and organs whose shape is more variable. All the considered acquisition reframings provide dynamic curves in agreement to each other’s, as illustrated with the 360 × 5s and 60 × 30s reframings in Fig. 6C and D. Of course, shorter time frames are more prone to noise, which induces fluctuations in the dynamic curves. Despite the larger time bins of the 60 × 30s reframing used to reduce the noise in the final image, the TACs still present some oscillations, similar to what is observed in dynamic planar results (Fig. 6A). Discontinuities are also visible in the curves in Fig. 5A around 3 min and in Fig. 5B around 6 min. These discontinuities might have some (unknown) physiological origin, but could also be produced by statistical Poisson fluctuations in the data. This noise in the TACs could be reduced by filtering the data points [15] or by performing some constrained reconstructions, i.e., that takes into account the fact that the activity in the images should change smoothly from one time bin to the next [2, 3].

The Starguide camera benefits from an improved energy resolution of about 6% at 140 keV thanks to its CZT detectors. Nevertheless, we have selected a 20% energy window for both the Starguide and the 2-head gamma camera acquisitions. This choice increases the Starguide sensitivity and lessen the noise impact on the reconstructed images, at the cost of more scatter counts. In the present study, this drawback is limited because of the small size of the pig. Further studies are required to evaluate the best energy window, i.e., the one providing the best compromise between the noise and scatter amounts in the measured counts.

The TACs of 2D and 3D dynamic acquisitions are nicely fitted by the adapted 2-compartment model (Eq. 4). From Fig. 6A and B, it appears that the 2 kidneys are not behaving the same way in the dynamic planar imaging, unlike for the dynamic SPECT imaging (Fig. 6C-D). It has already been shown that the kidney function is affected by anesthesia and bladder filling [9, 11]. As planar and SPECT acquisitions were performed on 2 consecutive days, the pig was subjected to 2 different anesthesia. Moreover, because of camera scheduling problems on the second day, the animal had to stay sedated longer before the scan was performed. This longer sedation not only impacted the kidney function but also the filling of the bladder at scan start.

From fit parameter’s values (Table 1), we see that, for the left kidney in dynamic planar imaging, there is almost no effect of the bladder filling (β is close to zero), but the kidney clearance is strongly slowed down (low value of k_o_). On the other hand, in the dynamic SPECT, both kidneys present a bladder effect indicated by a non-zero β. Interestingly the fit parameters for the right kidney present some similarities between 2D and 3D dynamic data: the uptake occurs at about the same speed (close k_i_ values) and a similar bladder effect is observed (β values of 0.154 and 0.130 for 2D and 3D TACs, respectively). The values of the fit parameters obtained for the 360 × 5s and 60 × 30s reframings (see Table 1) give an idea of the impact of the reframing, and indirectly of the noise in the reconstructed images, on the dynamic curves.

Regarding the kidney function evaluation, the results given in Table 2 show that dynamic SPECT imaging provides time-to-peak values of 2.2 and 2.7 min similar to dynamic planar imaging values of 2.6 and 3.0 min, for left and right kidneys, respectively. In their evaluation of ureteropelvic junction obstruction, Rowe et al. observed a mean time-to-peak of 50.9s and 722.5s for normal and obstructed swines, respectively [16]. Because of bladder filling, our values are larger than those for normal pigs but much lower than for obstructed cases. Similarly, our values for the kidney clearance halftimes of about 17 min and 6 min, for 2D and 3D acquisitions, respectively, are much longer than normal values of less than 5 min observed by Itano et al. [17]. Again, using different reframings of the acquisition produces variations of a few percent in the clearance halftimes as illustrated in Table 2. The time-to-peak values are less affected by the temporal reframing.

An advantage of the dynamic SPECT imaging is to enable regional analysis of the organs. This is simulated in the present study with upper and lower ROIs corresponding to kidney halves in the superior-inferior axis. Associated TACs are displayed in supplementary Fig. 1C and 1D, with fit parameters given in supplementary Table 1. The corresponding times-to-peaks and clearance halftimes are presented in supplementary Table 2. Differences were observed in TACs between upper and lower kidneys regions mainly due to the pelvic junction, included in the kidney lower half ROI, that increases the kidney total counts and the clearance halftime.

The present pilot study has some limitations: only 1 pig was included and unfortunately the animal preparation was not exactly the same for the SPECT and planar acquisitions, especially regarding the sedated duration before the scan. Introducing a catheter into the pig bladder would have lessen the bladder filling effect, at the cost of more intrusive acts and additional radioactive waste management. Regarding the theoretical modelling, simple assumptions were made to render the mathematical problem tractable. More complex approaches could be considered but introducing more parameters to fit only makes sense if the parameters are related to some physiological factors that can be estimated. It is always better to fit data with the minimum number of parameters to avoid overfitting.

## Conclusions

The present in vivo pilot study clearly shows that [^99m^Tc]Tc-MAG3 dynamic SPECT can be performed with CZT swivelling-detector ring cameras. Kidney’s TACs similar to standard dynamic planar imaging are obtained, from which functional parameters like time-to-peak and clearance halftime can be extracted. Further studies are required to evaluate the impact of acquisition and reconstruction parameters on the TACs and on the image quality.

### Electronic supplementary material

Below is the link to the electronic supplementary material.

Supplementary Material 1

## Data Availability

The data that support the findings of this study are available from the corresponding author upon reasonable request.

## Abbreviations

AC: Attenuation Corrected
CT: Computed Tomography
CZT: Cadmium Zinc Telluride
DICOM: Digital Imaging and COmmunications in Medicine
FOV: Field Of View
MLEM: Maximum Likelihood Expectation Maximization
NAC: Non-Attenuation Corrected
ROI: Region Of Interest
SPECT: Single Photon Emission Computed Tomography
TAC: Time-Activity Curve
