# Supplementary material for: Kidney dynamic SPECT acquisition on a CZT swiveling-detector ring camera: an in vivo pilot study
Source: BMC Med Imaging. 2024 Apr 22;24:94. doi: 10.1186/s12880-024-01271-y (PMC11036633; doi:10.1186/s12880-024-01271-y)
Supplement: Supplementary file 1 — Supplementary Material 1 [file 12880_2024_1271_MOESM1_ESM.docx]

**Supplementary materials for manuscript: Kidney dynamic SPECT acquisition on a CZT swiveling-detector ring camera: an in vivo pilot study**

Michel Hesse^1^ PhD, Florian Dupont^1^, Nizar Mourad^2^ PhD, Pavel Babczenko^2^, Gwen Beaurin^2^, Daela Xhema^2^, Eliano Bonaccorsi-Riani^2^ MD, PhD, François Jamar^1^, MD, PhD, Renaud Lhommel^1^ MD

1 Nuclear Medicine Department, Cliniques Universitaires Saint-Luc, Brussels, Belgium

2 Pôle de Chirurgie Expérimentale et Transplantation CHEX, Cliniques Universitaires Saint-Luc, Brussels, Belgium


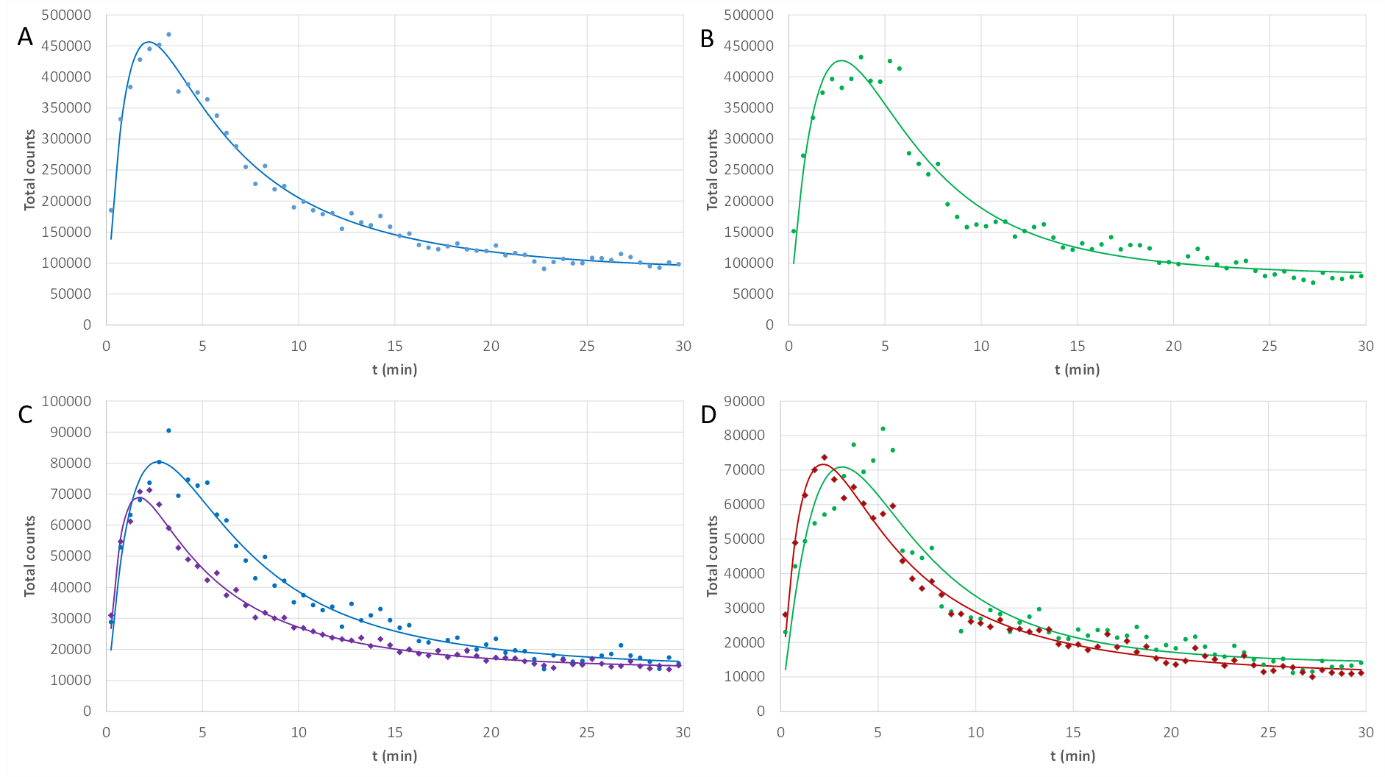
Supplementary Figure 1: TACs for the kidneys total counts from the 60x30s reframing of the dynamic SPECT acquisition. **A,B** TACs from the CTAC image for the left and right kidney, respectively. **C** TACs from the NAC image for the upper (purple diamonds) and lower (blue bullets) halves of the left kidney. **D** TACs from the NAC image for the upper (red diamonds) and lower (green bullets) halves of the right kidney. Full lines correspond to the theoretical fit of the data points.

Supplementary table 1: Parameters of the TACs fits of supplementary figure 1

| Left kidney | 3D CTAC 60x30s | 3D NAC 60x30s Kidney lower half | 3D NAC 60x30s Kidney upper half |
| --- | --- | --- | --- |
| A | 913651 | 244636 | 120494 |
| k_o_ | 0.236 | 0.295 | 0.252 |
| k_i_ | 0.935 | 0.655 | 1.367 |
| β | 0.100 | 0.104 | 0.116 |
| Right kidney | 3D CTAC 60x30s | 3D NAC 60x30s Kidney lower half | 3D NAC 60x30s Kidney upper half |
| A | 2491819 | 652500 | 163582 |
| k_o_ | 0.469 | 0.553 | 0.275 |
| k_i_ | 0.644 | 0.630 | 0.884 |
| β | 0.136 | 0.143 | 0.100 |

Supplementary table 1: Parameters of the TACs fits according to expression (4) for both kidneys for dynamic curves on supplementary figure 1

Supplementary table 2: Dynamic physiological parameters extracted from TACs of supplementary figure 1

| Left kidney | 3D CTAC 60x30s | 3D NAC 60x30s Kidney lower half | 3D NAC 60x30s Kidney upper half |
| --- | --- | --- | --- |
| Time to peak (min) | 2.2 | 2.7 | 1.7 |
| Clearance halftime (min) | 6.6 | 6.9 | 5.7 |
| Right kidney | 3D CTAC 60x30s | 3D NAC 60x30s Kidney lower half | 3D NAC 60x30s Kidney upper half |
| Time to peak (min) | 2.7 | 3.1 | 2.2 |
| Clearance halftime (min) | 6.2 | 6.3 | 5.8 |

Supplementary table 2: Time-to-peak and clearance halftime extracted from the fitted curves of the TACs corresponding to the CTAC and NAC images of the 60x30s reframings of the SPECT acquisition, and displayed on supplementary figure 1.
